# RETROSPECTIVE ANALYSIS OF MALLET FINGER TREATED WITH EXTENSION BLOCK FIXATION USING THE ISHIGURO TECHNIQUE

**DOI:** 10.1590/1413-785220263401e290553

**Published:** 2026-03-30

**Authors:** Alessandro Ayres Vianna, André Giacomazzi Martins, Santiago Martin Paredes Arcentales, Yussef Ali Abdouni, Antonio Carlos da Costa

**Affiliations:** 1 Irmandade da Santa Casa de Misericordia de Sao Paulo, Departamento de Ortopedia e Traumatologia "Pavilhao F. Simonsen", Sao Paulo, SP, Brazil.

**Keywords:** Fractures, Avulsion, Intra-Articular Fractures, Osteosynthesis, Fracture, Fratura Avulsão, Fraturas Intra-articulares, Osteossíntese

## Abstract

**Objective:**

This study aimed to evaluate the outcomes of the Ishiguro technique in the treatment of flexion deformity of the interphalangeal distal (IFD) joint, known as bony mallet finger (BMF) Albertoni Type C2, addressing a common condition that may cause functional impairment if not adequately treated.

**Methods:**

All cases of patients with Albertoni Type C2 bony mallet finger (BMF) who underwent the Ishiguro method between March 2018 and February 2023 were included, with a minimum postoperative follow-up of five months; cases of patients who did not complete the minimum five-month postoperative follow-up were excluded. Epidemiological data (age, sex, dominance) and fracture-related information (affected finger, fragment size, time from trauma to surgery, follow-up duration) were analyzed. Outcomes included anatomic reduction, union, return to activities, pain, flexion deficit, passive extension of the affected finger, passive extension of the contralateral finger, and Crawford criteria.

**Results:**

None of the analyzed characteristics—such as age, affected finger, interval between trauma and surgery, fragment size, and union—significantly influenced anatomic reduction (p > 0.05). The Crawford scale, as well as anatomic reduction, showed no significant impact (p > 0.05). However, all patients with positive results on the Crawford scale demonstrated union (p = 0.063).

**Conclusion:**

In our series, more than half of the patients achieved good or excellent results, predominantly characterized by anatomic reduction. No significant associations were identified with variables such as age, affected finger, time from trauma to surgery, fragment size, bone union, or anatomic reduction. These findings suggest that the Ishiguro technique may be effective in the treatment of BMF Albertoni Type C2. ***Level of Evidence III; Retrospective Study***.

## RESUMO

**Objetivo:**

este estudo teve como propósito avaliar os resultados da técnica de Ishiguro no tratamento da deformidade em flexão da articulação interfalângica distal (IFD), conhecida como dedo em martelo ósseo (DMO) do Tipo C2 de Albertoni, visando abordar uma condição comum que pode causar déficit funcional se não tratada adequadamente.

**Método:**

foram incluídos todos os casos de pacientes com dedo em martelo ósseo (DMO) do Tipo C2 de Albertoni que foram submetidos ao método de Ishiguro entre março de 2018 e fevereiro de 2023, com acompanhamento mínimo de cinco meses pós-cirurgia; excluídos casos de pacientes que não completaram o acompanhamento mínimo de cinco meses pós-cirurgia. Dados epidemiológicos (idade, sexo, dominância) e informações sobre a fratura (dedo afetado, tamanho do fragmento, tempo entre trauma e cirurgia, tempo de seguimento) foram analisados. Os desfechos incluíram redução anatômica, consolidação, retorno às atividades, dor, déficit de flexão, extensão passiva do dedo afetado, extensão passiva do dedo contralateral e critérios de Crawford.

**Resultado:**

nenhuma característica analisada, como idade, dedo afetado, intervalo entre trauma e cirurgia, tamanho do fragmento e consolidação, influenciou significativamente a redução anatômica (p > 0,05). A escala de Crawford, assim como a redução anatômica, não apresentou impacto significativo (p > 0,05). Contudo, todos os pacientes com resultados positivos na escala de Crawford exibiram consolidação (p = 0,063).

**Conclusão:**

na nossa série, mais da metade dos pacientes obteve resultados bons ou excelentes, predominantemente caracterizados por redução anatômica. Não foram identificadas relações significativas com variáveis como idade, dedo acometido, tempo entre trauma e cirurgia, tamanho do fragmento, consolidação óssea ou redução anatômica. Estes achados sugerem que a técnica de Ishiguro pode ser eficaz no tratamento do DMO Tipo C2 de Albertoni. ***Nível de Evidência III; Estudo Retrospectivo***.

## INTRODUCTION

The flexion deformity of the interphalangeal distal joint (IFD), known as the hammer finger, is common and can cause functional deficit if not properly treated.^1,2^ The diagnosis of the deformity is clinical, but radiography is necessary to differentiate between tendon lesions and bone lesions with a fracture of the dorsal margin of the base of the distal phalanges.^3^

Although conservative treatment with thistles shows good results in some cases, the maintenance of the joint step can lead to post-traumatic arthrosis and pain. Thus, when there is compromising at least one third of the joint surface of the distal phalanx or subluxation of IFD^4^, there is the need for anatomical reduction, which can be obtained open or closed, followed by fixation with Kirschner wires, pull-out suture,^4,5^ tension band,^6^ mini interfragmentary screw^7^, or even a hook plate.

**Figure 1:**
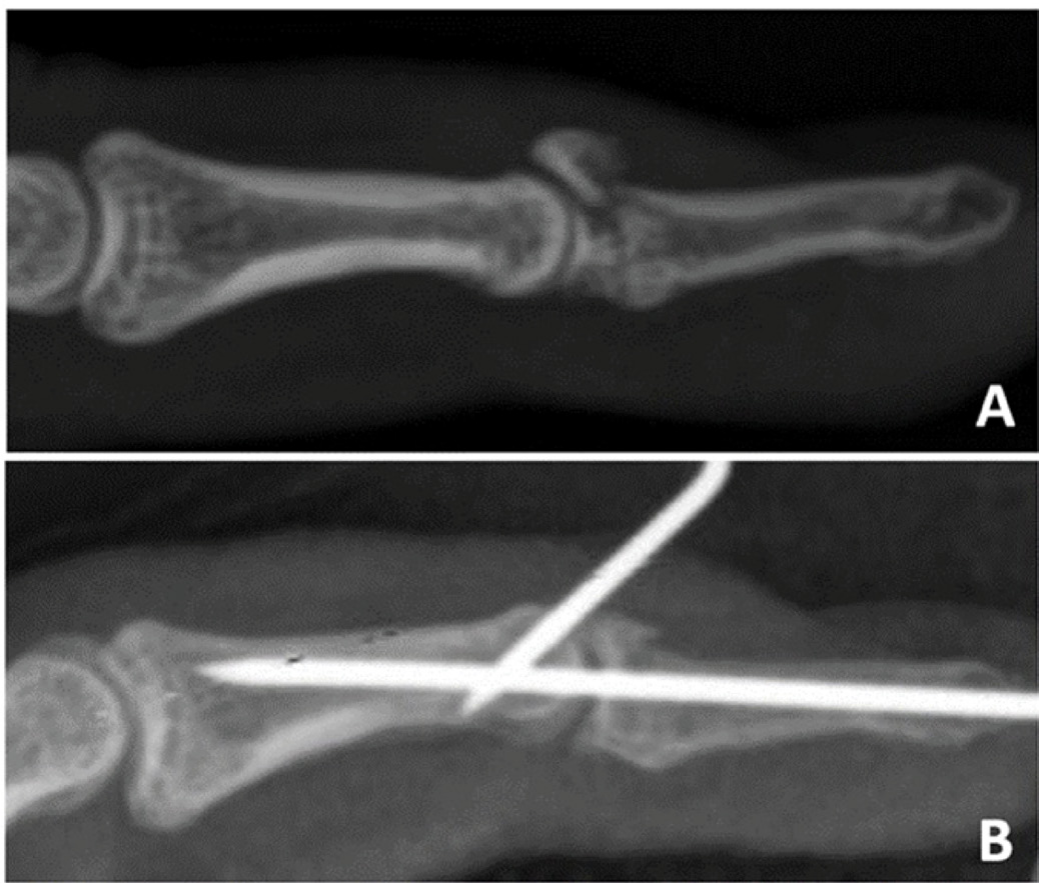
BMF lateral X-ray (A) and after fixation by Ishiguro method (B).

Source: Author.

The techniques of reduction and open fixation, in general, present some risks, which include infection, nail deformity,^8,9^ hypertrophic scar formation,^10^ prominence of the synthesized material,^7,9^ comminution of the bone fragment during the fixation attempt,^7^ etc. Thus, the percutaneous Kirschner wire is frequently employed in fracture osteosynthesis, for which the most diverse configurations have been described.^11-14^ Ishiguro et al.^15^ introduced a method for closed finger reduction in bony mallet finger (BMF), in which the extension of IFD with Kirschner wire is performed (Figure 1). Since then, some authors have modified the configuration of the wires,^16,17^ while retaining the fixation concept of the original technique.^15^

Although it is a technique that has become popular since its publication, because it is considered simple, fast, easily reproducible, and with fewer complications than the open methods,^18^ few papers in the literature evaluated the results of Ishiguro et al. Inoue et al. reported success rates of 84% in closed surgical treatments.^19^ Lee and Hyun observed that there was no statistical difference in the results when comparing closed and open techniques, but in the open-air reduction group 22% of complications of skin or nail bed, sensitivity at the site of incision, plus longer surgical time were observed.^20^

This work aims to evaluate the results of the technique of Ishiguro et al,^15^ using clinical and radiographic parameters, of BMF Type C2 of Albertoni et al.^21^

## MATERIALS AND METHODS

**Figure 2:**
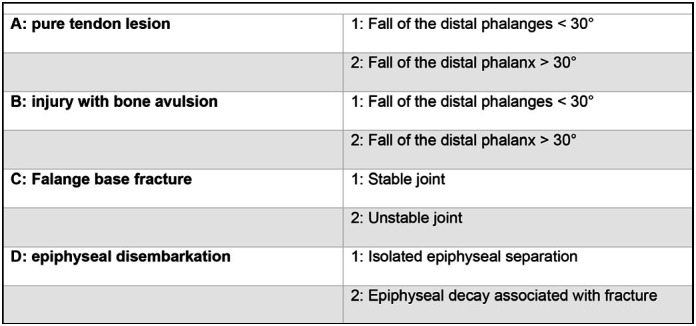
Classification of Albertoni et al.^21^

For the purposes of this study, 16 fingers were evaluated from 16 patients with BMF Type C2 of Albertoni et al.^21^ (Figure 2) treated by Ishiguro et al. Method^15^ between March 2018 and February 2023, with a minimum follow-up time of five months after surgery, with the approval of the ethics and research committee (opinion 5.438.893). All participants signed the Free and Informed Consent Clause, with the approval of the Ethics and Research Committee (CAAE 44575121.9.0000.5479)

**Figure 3:**
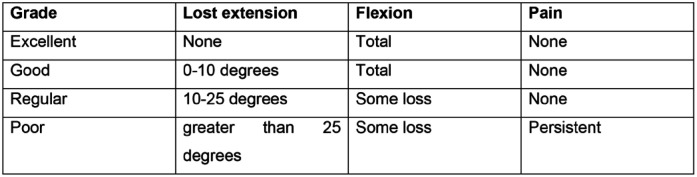
Crawford's criteria for the result of the hammer finger treatment.

We evaluated epidemiological data (age, gender, dominance), fractural data (affected finger, fragment size, time between trauma and surgery and follow-up time) (Table 1) and outcomes (anatomical reduction, consolidation, return to activities, pain, flexion deficit, passive and passive extension of the affected finger, passive and passive extension of the corresponding contralateral finger and Crawford criteria.^22^ (Figure 3)

**Table 1:** Descriptive statistics of the variables age, gender, dominance, affected finger, fragment size, time between trauma and surgery, and follow-up time.

Variable		Description
		(N = 16)
**Age (years)**		
Average ± SD		41.7 ± 12.8
median (p25; p75)		44 (33; 47)
**Gender, n (%)**		
	Female	8 (50)
	Male	8 (50)
**Dominance, n (%)**		
	Right(a)	16 (100)
**Finger, n (%)**		
	Left index	2 (12.5)
	middle left	2 (12.5)
	right ring	1 (6.3)
	left ring	4 (25)
	little right	3 (18.8)
	little left	4 (25)
**Fragment size (%)**		
	Average ± SD	53.8 ± 8.2
	median (p25; p75)	53 (50; 60.8)
**Time between trauma and surgery (days)**	
	Average ± SD	9.6 ± 10.3
	median (p25; p75)	7.5 (2.3; 14)
**Tracking time (months)**		
	Average ± SD	11.1 ± 5.1
	median (p25; p75)	11 (6; 13.8)

Qualitative characteristics evaluated in all patients using absolute and relative frequencies were described, and quantitative characteristics were described for all patients using summary measurements (average, standard deviation, median, and quartile).^23^

**Figure 4:**
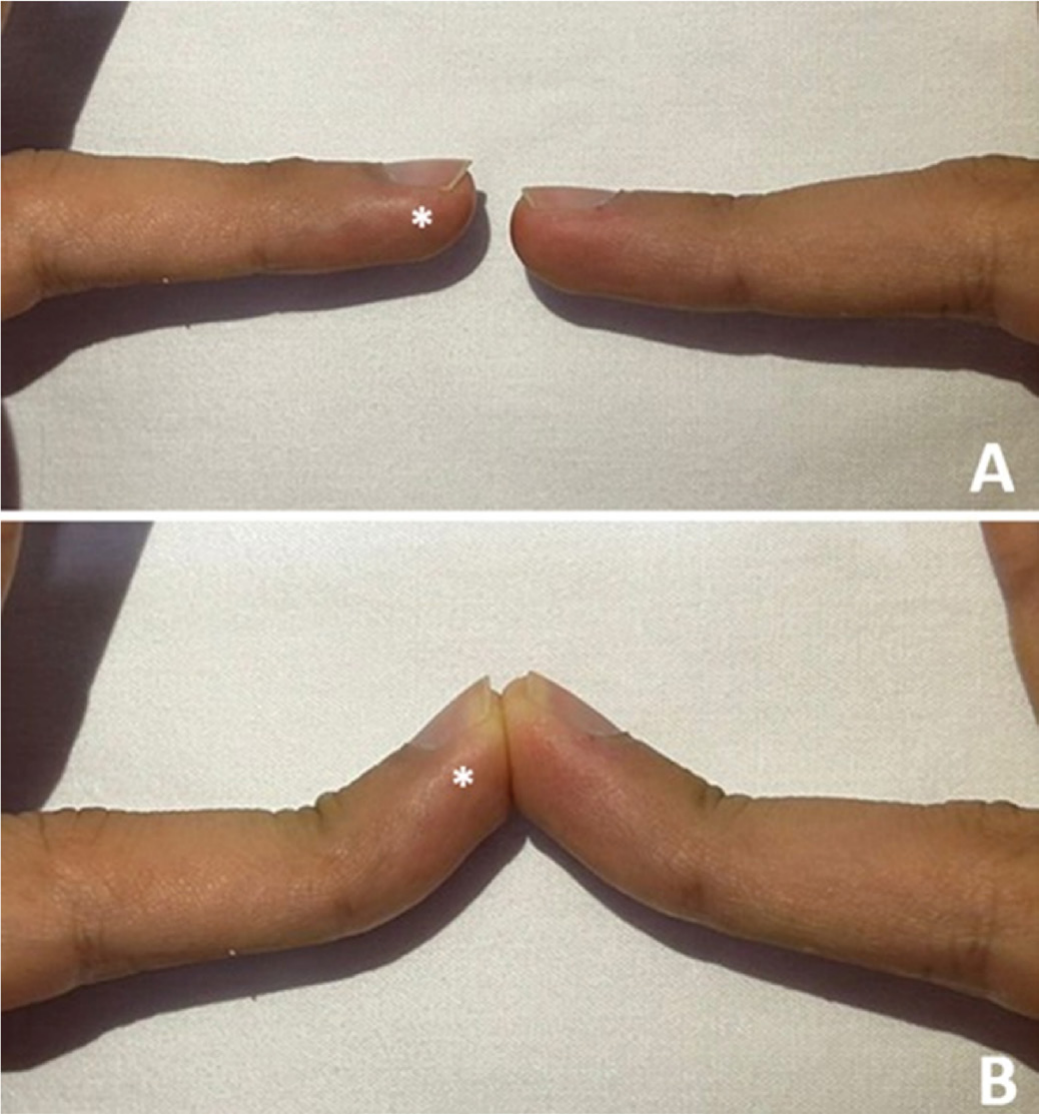
BMF image after 2 months of rehabilitation, comparing active extension (A) and passive extension (B) of the affected finger (*) with the contralateral finger.

Source: Author.

The good and excellent results, according to the Crawford criteria, were grouped, as well as the bad and regular ones. Still in relation to the Crawford criteria, we calculated the movement deficit of the IFD relative to the counter-side finger. (Figure 4)

Success in reduction and the best result on the Crawford scale were described according to the qualitative characteristics of interest, and the association of these parameters was verified with the use of Fisher Exact Tests or Tests of the Ratio of Probability Similarities. The ages and size of the fragment were described according to the outcomes and compared with the use of non-pairing t-Student tests, since the time between trauma and surgery was described according to the outcomes and compared with the use of Mann-Whitney tests, because the distribution of the data did not present normal distribution.^23^ For all variables the odds ratios (OR) not adjusted with the respective intervals were estimated with 95% confidence using simple logistical regressions.^24^

The analysis was performed using the IBM-SPSS *software for Windows* version 22.0, and the data tabulation was performed using the Microsoft Excel 2013 software. The tests were conducted with a significance level of 5%.

## RESULTS

None of the characteristics of interest (age, affected finger, time between trauma and surgery, fragment size, and consolidation) statistically influenced the anatomical reduction (p > 0.05), and the results found are described in Table 2.

**Table 2:** Description of the anatomical reduction according to the characteristics of interest (age, affected finger, time between trauma and surgery, fragment size, and consolidation) and the result of the unadjusted analyses.

Variable					OR	CI (95%)		p
			**No (N = 4)**	**Yes (N = 12)**		**Lower**	**Upper**	
Age (years)					0.87	0.73	1.04	0.068**
Average ± SD			51.8 ± 15	38.3 ± 10.7				
Median (p25; p75)			54.5 (36.8; 64)	44 (29.3; 45.5)				
		Gender. n (%)						>0.999
		Female	2 (25)	6 (75)		1.00		
		Male	2 (25)	6 (75)	1.00	0.10	9.61	
		Finger. n (%)						0.129#
		Index	0 (0)	2 (100)			1.00	
		Middle	1 (50)	1 (50)			&	
		Ring	0 (0)	5 (100)			&	
		Little	3 (42.9)	4 (57.1)			&	
Time between trauma and surgery (days)					1.01	0.90	1.14	0.953£
		Average ± SD	8.8 ± 8.3	9.9 ± 11.2				
		Median (p25; p75)	7.5 (1.8; 17)	7 (2.3; 14)				
		Fragment Size (%)			1.04	0.90	1.20	0.639**
		Average ± SD	52 ± 9.2	54.3 ± 8.2				
		Median (p25; p75)	53.5 (42.5; 60)	53 (50; 61.5)				
		Consolidated. n (%)						0.136
		No	2 (66.7)	1 (33.3)	1.00			
		Yes	2 (15.4)	11 (84.6)	11.00	0.65	187.17	

Exact Fisher test;

# Test of the likelihood ratio;

** Unmatched t-Student test;

£ Mann-Whitney test; & Can't be estimated.

From Table 3, it is shown that, as well as the anatomical reduction, the Crawford scale did not present a statistically significant influence on any characteristic of interest evaluated (p > 0.05), but all patients who presented positive results on the Crawford scale presented consolidation (p = 0.063).

**Table 3:** Description of the Crawford scale according to the characteristics of interest (age, affected finger, time between trauma and surgery, fragment size, consolidation, and anatomical reduction) and the result of the unadjusted analyses.

Variable		Crawford		OR	CI (95%)		p
		**Bad/Regular (N = 7)**	**Good/Excellent (N = 9)**		**Lower**	**Superior**	
Age (years)				0.97	0.89	1.05	0.470**
Average ± SD		44.4 ± 15.2	39.6 ± 11.1				
median (p25; p75)		44 (39; 54)	44 (28.5; 47)				
Gender. n (%)							>0.999
	Female	4 (50)	4 (50)	1.00			
	Male	3 (37.5)	5 (62.5)	1.67	0.23	12.22	
Finger. n (%)							0.204#
	Index	2 (100)	0 (0)	1.00			
	Middle	1 (50)	1 (50)	&			
	Ring	1 (20)	4 (80)	&			
	Little	3 (42.9)	4 (57.1)	&			
Time between trauma and surgery (days)				1.06	0.93	1.20	0.252£
Average ± SD		7 ± 8.4	11.7 ± 11.7				
Median (p25; p75)		2 (1; 16)	8 (5.5; 14)				
Fragment Size (%)				0.95	0.84	1.08	0.490**
Average ± SD		55.4 ± 7.6	52.4 ± 8.9				
Median (p25; p75)		57 (50; 62)	50 (45; 60.5)				
Consolidated. n (%)							0.063
	No	3 (100)	0 (0)	1.00			
	Yes	4 (30.8)	9 (69.2)	&			
Anatomic reduction. n (%)							>0.999
	No	2 (50)	2 (50)	1.00			
	Yes	5 (41.7)	7 (58.3)	1.40	0.14	13.57	

Exact Fisher test;

# Test of the likelihood ratio;

** Unmatched t-Student test;

£ Mann-Whitney test; & Can't be estimated.

## DISCUSSION

There is no consensus on whether the anatomical reduction achieved through surgical treatment is essential for the patient to have a good functional recovery. When there is subluxation or fracture affecting more than one-third of the joint surface, most authors suggest that the best treatment is surgical with reduction of the avulsed fragment.^1,4,5,14,16,18,19,25,26^ In these cases, the technique of percutaneous fixation with extension blockage is a quick and easy technique. However, there is little information on the long-term functional outcome of patients undergoing the technique described by Ishiguro et al.^15^

In our study, we analyzed the anatomical reduction of the fracture with age, gender, affected finger, time between trauma and surgery, fragment size, and consolidation; and none of the characteristics evaluated presented a relationship with statistical significance.

We believe that gender, dominance, and affected finger could vary due to patient demand for their activities. Male patients in our service tend to perform tasks that require greater physical strength, and female patients, on the other hand, have a demand for tasks that require greater precision. Similarly, the dominant limb and the affected finger influence the representativity that the injury region exerts in the patient's daily work and exercises.

The average age of the patients who presented anatomical reduction was 38.3 years, being lower than the average of the others of 51.8 years. This could suggest that the lower age predisposes to a better postoperative radiographic alignment immediately, and may be due to better bone quality in younger patients, but due to the low number of cases, we cannot confirm such a statement.

Another factor evaluated was the time between the patient's trauma and the surgical fixation. The process of consolidation begins at the initial moment with the formation of a hematoma and subsequent organization in a bone callus.^27^ It was believed that the delay in treatment caused the organization of fibrosis and callus in the area of the focus of fracture and the retraction of the tendon^28^ to make it difficult to reduce the fragment. However, in this series, we did not find a relationship between the result and the delay in treatment, as one would expect; on the contrary, the patient with the longest time between injury and surgery developed an excellent result.

Taking into account the follow-up time, after removal of the wires, patients needed rehabilitation to improve finger mobility. The minimum follow-up time was five months, which was sufficient to observe the result after stabilizing the possible function gain. On the other hand, we cannot infer whether these lesions will evolve with post-traumatic arthrosis as a late complication, leading to long-term impairment of function.

**Figure 5:**
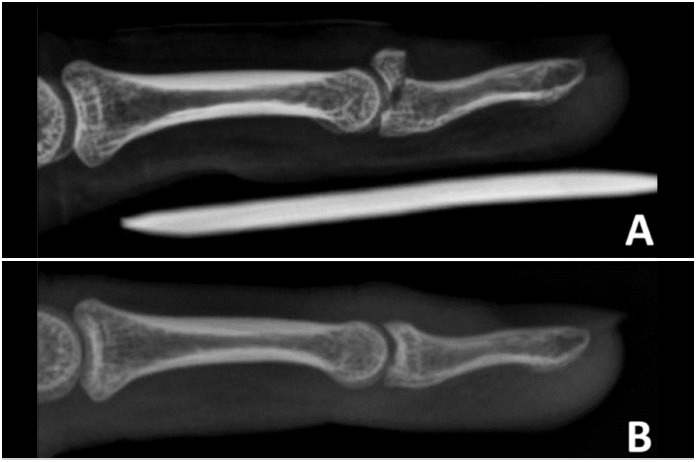
Initial lateral radiography (A) and after 3 months of surgery (B).

Source: Author.

Because it is a bone lesion, following the principles of the treatment of fractures, when there is joint impairment, anatomical reduction and rigid fixation are necessary.^29^ Larger bone fragments are most often better reduced due to ease in intraoperative manipulation; however, the cases with a higher percentage of affected joints were not associated with better reduction. In twelve cases, anatomical reduction was achieved, and in thirteen cases, there was consolidation (Figure 5); however, we did not observe a correlation between these two variables as expected, since two cases that did not present anatomical reduction had consolidation, and one reduced anatomical case did not show consolidation.

The patients with better results, according to the Crawford criteria, classified as good and excellent, all presented consolidation of the fracture. Among these factors, what led our study patients to be classified as lower results was predominantly the deficit of extension, which corroborates that the most statistically significant factor is the consolidation of the fragment. The hammer finger bone fragment is adhered to the terminal extensor tendon,^30^ and its non-consolidation suggests that the action of this tendon on the distal phalanges will be impaired. Thus, more than the reduction achieved intraoperatively, it is conceivable that fracture consolidation has greater relevance for long-term functional outcome.

Due to the limited number of cases, more studies are needed to determine which factors would actually have the greatest influence on long-term functional outcomes.

## CONCLUSION

We concluded that, in our series, just over half showed good or excellent results, that the majority of cases had anatomical reduction, and that there was no relationship with age, affected finger, time between trauma and surgery, fragment size, presence of bone consolidation, or anatomical reduction.

## Data Availability

The contents underlying the research are available in the manuscript.
